# Supplementary material for: Association of KMT2C Genetic Variants with the Clinicopathologic Development of Oral Cancer
Source: Int J Environ Res Public Health. 2022 Mar 27;19(7):3974. doi: 10.3390/ijerph19073974 (PMC8997509; doi:10.3390/ijerph19073974)
Supplement: Supplementary file 1 [file ijerph-19-03974-s001.zip › KMTC2-OSCC-IJERPH-Supplementary Table S1-20220127.pdf]

Supplementary Table S1. Clinical statuses and KMT2C rs4725443 genotype frequencies in cases of OSCC group among 233 betel nuts chewing and 251 cigarette smoking.

**Variable**	**KMT2C (rs4725443)**					
	**Betel nut chewing (N=233)**			**Cigarette smoking (N=251)**		
	**TT (%) (N = 147)**	**TC + CC (%) (N = 86)**	***p* Value**	**TT (%) (N = 162)**	**TC + CC (%) (N = 89)**	***p* Value**
**Clinical stage**						
Stage I/II	87 (59.2%)	46 (53.5%)	p = 0.397	101 (62.3%)	51 (57.3%)	*p* = 0.435
Stage III/IV	60 (40.8%)	40 (45.5%)		61 (37.7%)	38 (42.7%)	
**Tumor size**						
T1 + T2	99 (67.3%)	54 (62.8%)	p = 0.480	115 (71.0%)	61 (68.5%)	*p* = 0.685
T3 + T4	48 (32.7%)	32 (37.2%)		47 (29.0%)	28 (31.5%)	
**Lymph node metastasis**						
No	111 (75.5%)	63 (73.3%)	p = 0.703	125 (77.2%)	67 (75.3%)	*p* = 0.737
Yes	36 (24.5%)	23 (26.7%)		37 (22.8%)	22 (24.7%)	
**Distant metastasis**						
No	138 (93.9%)	82 (95.3%)	p = 0.638	151 (93.2%)	84 (94.4%)	*p* = 0.717
Yes	9 (6.1%)	4 (4.7%)		11 (6.8%)	5 (5.6%)	
**Cell differentiation**						
Well	23 (15.6%)	19 (22.1%)	p = 0.219	26 (16.0%)	19 (21.3%)	*p* = 0.297
Moderate/poor	124 (84.4%)	67 (77.9%)		136 (84.0%)	70 (78.7%)	

N: number.
